# A simple nomogram for sample size for estimating sensitivity and specificity of medical tests

**DOI:** 10.4103/0301-4738.71699

**Published:** 2010

**Authors:** Rajeev Kumar Malhotra, A Indrayan

**Affiliations:** Department of Biostatistics and Medical Informatics, University College of Medical Sciences, New Delhi – 110 095, India

**Keywords:** Nomogram, sample size, sensitivity, specificity

## Abstract

Sensitivity and specificity measure inherent validity of a diagnostic test against a gold standard. Researchers develop new diagnostic methods to reduce the cost, risk, invasiveness, and time. Adequate sample size is a must to precisely estimate the validity of a diagnostic test. In practice, researchers generally decide about the sample size arbitrarily either at their convenience, or from the previous literature. We have devised a simple nomogram that yields statistically valid sample size for anticipated sensitivity or anticipated specificity. MS Excel version 2007 was used to derive the values required to plot the nomogram using varying absolute precision, known prevalence of disease, and 95% confidence level using the formula already available in the literature. The nomogram plot was obtained by suitably arranging the lines and distances to conform to this formula. This nomogram could be easily used to determine the sample size for estimating the sensitivity or specificity of a diagnostic test with required precision and 95% confidence level. Sample size at 90% and 99% confidence level, respectively, can also be obtained by just multiplying 0.70 and 1.75 with the number obtained for the 95% confidence level. A nomogram instantly provides the required number of subjects by just moving the ruler and can be repeatedly used without redoing the calculations. This can also be applied for reverse calculations. This nomogram is not applicable for testing of the hypothesis set-up and is applicable only when both diagnostic test and gold standard results have a dichotomous category.

Sensitivity and specificity are two components that measure the inherent validity of a diagnostic test compared to the gold standard; a valid test would not only correctly detect the presence of disease but also correctly detect the absence of the disease in subjects with and without disease, respectively. Sensitivity and specificity are useful measures when the established gold standard is difficult to adopt in practice. For example, diagnosis of pancreatic carcinoma can be confirmed only by laprotomy for alive or by autopsy for dead patients. Sometimes the gold standard is expensive, less widely available, more invasive, riskier, and takes more time to produce results. Such issues compel researchers to develop new diagnostic methods as surrogate to the gold standard.

An adequate sample size is needed to ensure that the study will yield estimate of the sensitivity and specificity with acceptable precision—smaller sample size produces imprecise estimate, and unduly large sample is wastage of resources especially when the new method is expensive. Furthermore, the prevalence of disease was included in the sample size formula by Buderer, because the sample size without considering the prevalence would be adequate either for sensitivity or for specificity but not for both.[1]

In practice, researchers generally decide a sample size for validating a new diagnostic test arbitrarily or at their convenience or use the previous literature. A study was conducted by Bochmann in five highest impact factor ophthalmology journals to assess the sample size calculation in diagnostic accuracy articles published in 2005 and found only 1 out of 40 studies reporting the sample size calculation before initiating the study.[2] This may be due to reluctance in using a mathematical formula or computer software. Buderer provides the sample size tables for sensitivity and specificity but they are only for the 10% precision level. Carley *et al*. have provided nomograms but they are separate for sensitivity and specificity. They derived them only for the 95% level of confidence; too many lines and curves make their nomogram complex to read.[3]

A nomogram is a chart consisting of three or more lines or curves so arranged that the required reading can be quickly made by just moving the ruler. They are still very popular in spite of the availability of computer. One of the main attractions is that a nomogram can be carried anywhere since it is just a piece of paper and can be repeatedly used without redoing the calculations. Various nomograms have been devised such as to calculate the sample size in diagnostic studies, to find the number of clusters required for estimating the prevalence rate in single-stage cluster-sample survey, and to find the number needed to treat in a therapeutic trial against values of absolute risk in the absence of treatment.[3–5]

We have devised a relatively very simple nomogram to read the sample size for anticipated sensitivity and specificity using the formula described by Buderer.[1] This guides the researchers about the adequate sample size to achieve specified absolute precision. The estimated prevalence of disease and confidence level 100(1 – α)% are required. The features of this nomogram are as follows: (i) a single nomogram can be used to read the sample size for both sensitivity and specificity, (ii) it is based on simple lines instead of curves, (iii) it is easy to read by just moving the ruler from one point to another, (iv) the sample size for the 95% confidence level is directly available and one can calculate the sample size for 99% and 90% levels of confidence just multiplying by 1.75 and 0.70, respectively to sample size obtained by using 95% confidence level, and (iv) the sample size can be obtained for any precision level with minor calculations.

## Materials and Methods

Sample size at the required absolute precision level for sensitivity and specificity can be calculated by Buderer’s formula:[1]

$$\mathrm{Sample}\;\mathrm{size}\;\left(n\right)\;\mathrm{based}\;\mathrm{on}\;\mathrm{specificity}=\frac{Z_{1-\alpha /2}^{2}\;\times \;S_{N}\;\times \left(1-S_{N}\right)}{L^{2}\;\times \;\mathrm{Prevalence}},\mathrm{and}\;$$

$$\mathrm{sample}\;\mathrm{size}\;\left(n\right)\;\mathrm{based}\;\mathrm{on}\;\mathrm{specificity}=\frac{Z_{1-\alpha /2}^{2}\;\times \;S_{P}\;\times \left(1-S_{P}\right)}{L^{2}\;\times \;\left(1-\mathrm{Prevalence}\right)},\;$$

where *n* = required sample size,

*S_N_* = anticipated sensitivity,

*S_P_* = anticipated specificity,

*α* = size of the critical region (1 – *α* is the confidence level),

*z*_1-α/2_ = standard normal deviate corresponding to the specified size of the critical region (α), and

*L* = absolute precision desired on either side (half-width of the confidence interval) of sensitivity or specificity.

**Figure 1:**
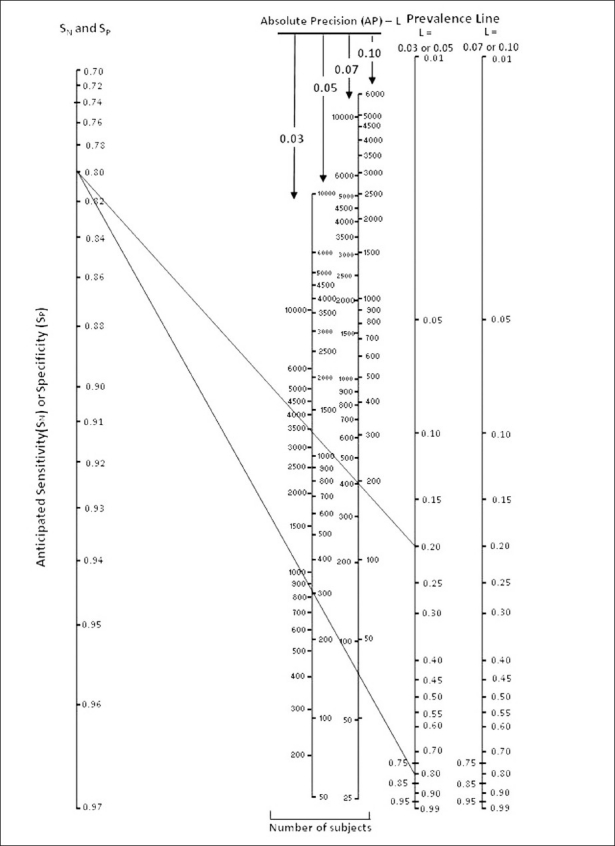
Nomogram for the sample size for anticipated sensitivity/specificity, and estimated prevalence

The procedure to construct a nomogram is described by Adam and Molnar.[6,7] Our nomogram is depicted in Fig. 1. This was created in MS Excel. This nomogram is for the 95% confidence level and consists of five parallel lines. The first line depicts anticipated sensitivity or specificity of the diagnostic test that can vary from 0.70 to 0.97. A test with anticipated sensitivity or specificity less than 0.70 may not be worthy of investigations. The minimum value of *L* on either side of anticipated sensitivity or specificity is taken as 0.03. The second line depicts the number of subjects required at 0.03 and 0.05 absolute precision and the third line depicts the number of subjects for 0.07 and 0.10 absolute precision. Fourth and fifth lines are prevalence lines and represent the expected prevalence of disease; the fourth line is to be used for *L* = 0.03 or 0.05 and the fifth for *L* = 0.07 or 0.10.

## Result

To find the number of subjects required for estimating sensitivity, place a ruler joining the anticipated sensitivity with expected prevalence and read the number of subjects where the ruler cuts the corresponding line of the number of subjects with required absolute precision. One should choose anticipated sensitivity such that after adding the required precision it does not exceed 1. For example, when anticipated sensitivity is 0.96, a researcher cannot select required precision to be more than 0.04.

Suppose the researcher selects anticipated sensitivity S*_N_* = 0.80, precision = 0.03 with 95% confidence level (two-tailed), i.e., S*_N_* can be from 0.77 to 0.83, and expected prevalence = 0.20. Place a ruler joining the point 0.80 on the anticipated sensitivity/specificity line to point 0.20 on the estimated prevalence line of 0.03 absolute precision and read the required sample size from the number of subjects line of 0.03 absolute precision. In our example, the number of subjects required is nearly 3450 as shown in Fig. 1. By formula, the exact value is 3415–a difference of nearly 1%. This can happen with any nomogram.

To find the required sample size for estimating specificity, first subtract the expected prevalence from 1 and place the ruler joining the anticipated specificity to (1 – prevalence) value on the prevalence line of required precision. For example, if *S_P_*= 0.80, precision = 0.05 with 95% confidence level, and prevalence is 0.20, join the point *S_p_* = 0.80 with the point (1 – 0.20) = 0.80, on the prevalence line of 0.05 absolute precision, and read the sample size from the number of subjects line for 0.05 absolute precision This is nearly 300. By calculation, the exact value is 308. Now the difference is 3%.

The final sample size depends on the interest of the researcher. If sensitivity and specificity are equally important for the study, determine the sample size for both sensitivity and specificity, separately. The final sample size of the study would be the larger of these two. But sometimes the researcher is interested more in sensitivity than specificity. In that case, the final sample size would be based on the sensitivity only. In addition, there are other considerations such as nonresponse and subgroup analysis.[8]

It is easily seen in the formula that the number of subjects is exactly four times when the length of *L* is halved, and one-fourth when the length of *L* is doubled, provided other values remain same. Following expression can be used to obtain the number of subjects needed for any precision level *L*_1_

(1) $$n_{1}\;=\;n_{0}\;\times \;{\left(\frac{L_{0}}{L_{1}}\right)}^{2}$$

where *n*_0_= sample size at precision level *L*_0_from the nomogram where *L*_0_may be 0.03, 0.05, 0.07, and 0.10 as depicted in our nomogram and *n*_1_= sample size at precision level *L*_1_; *L*_1_ may be any other acceptable precision level. Thus this nomogram can in fact be used for any precision level with minor calculation as envisaged in equation (1). Similarly the researcher can also use the nomogram for 99% and 90% confidence levels. To find the sample size for 99% and 90% confidence levels, first read the number of subjects required assuming the 95% confidence level and then multiply it with 1.75 for the 99% confidence level and 0.70 for the 90% confidence level. This is the ratio of the square of the standard normal deviate for the required confidence level 100(1 – *α*)% to the standard normal deviate for the 95% confidence level.

To validate the nomogram, various parameter combinations such as anticipated sensitivity/specificity, and prevalence of the disease were randomly selected. The exact sample size was calculated by the formula while at the same time independently second author determined the sample size from a nomogram for these randomly selected combinations of parameters. The percentage error was calculated (Tables 1 and 2 in Appendix). The percentage error is higher when the sample size is small; for instance, the exact sample size for specificity = 0.97, prevalence = 0.60, and absolute precision = 0.10 is 28 while the nomogram shows this as 30 [Table 2]. The difference of 2 is small although percentage is 7.14%. Otherwise the sample size is within 5% of the exact value. As already stated, this kind of minor approximation is inevitable with any nomogram as it simplifies the process.

## Discussion

A nomogram depicts the mathematical relationship among various parameters and is simple to use without redoing the calculations. Our nomogram has four parameters—anticipated sensitivity/specificity, number of subjects, absolute precision level, and expected prevalence of disease. Researchers can also use this nomogram for reverse calculation. If any of these three parameters are known, the fourth parameter can be obtained. This nomogram does not incorporate Type II error; thus this cannot be used for testing the hypothesis on sensitivity/specificity.

One of the main limitations of any nomogram is reading accuracy. In place of 465, one might read 460 from the line but this minor deviation may not be important in practice. This nomogram is applicable only when both the new diagnostic test and gold standard provide result in a dichotomous category such as test+ and test−. Thus this is not applicable when the gold standard is dichotomous and the new diagnostic test is ordinal or continuous, or vice versa.

## Appendix

**Table 1:** Validation table for the sample size for estimating sensitivity with the 95% confidence level

Sensitivity	Prevalence	Exact sample size by formula				Sample size by nomogram				Difference in percentage			
		Absolute precision				Absolute precision				Absolute precision			
		0.03	0.05	0.07	0.10	0.03	0.05	0.07	0.10	0.03	0.05	0.07	0.10
0.70	`0.20	4482	1613	823	403	4500	1610	820	400	+0.40	−0.19	−0.36	−0.74
0.73	0.20	4207	1514	773	379	4125	1500	795	380	−1.95	−0.92	+2.85	+0.26
0.80	0.40	1707	615	314	154	1700	620	312	160	−.41	+0.81	−0.64	+3.90
0.84	0.10	5737	2065	1054	516	5800	2050	1070	510	+1.10	−0.73	+1.52	−1.16
0.85	0.30	1814	653	333	163	1875	660	330	170	+3.36	+1.07	−0.90	+4.29
0.90	0.50	768	277	141	69	790	285	148	70	+2.86	+2.89	+4.96	+1.45
0.93	0.45	618	222	113	56	625	225	115	55	+1.13	+1.35	+1.77	−1.79
0.97	0.25	497	179	91	45	490	185	90	44	−1.4 1	+3.35	−1.10	−2.22

**Table 2:** Validation table for the sample size for estimating specificity with the 95% confidence level

Specificity	Prevalence	Exact sample size by formula				Sample size by nomogram				Difference in percentage			
		Absolute precision				Absolute precision				Absolute precision			
		0.03	0.05	0.07	0.10	0.03	0.05	0.07	0.10	0.03	0.05	0.07	0.10
0.70	0.15	1055	380	194	95	1050	375	197	95	−0.47	−1.32	+1.55	0.00
0.73	0.20	1052	379	193	95	1050	375	197	95	−0.19	−1.06	+2.07	0.00
0.80	0.25	911	328	167	82	900	320	175	82	−1.21	−2.44	+4.79	0.00
0.84	0.35	883	318	162	79	900	320	170	82	+1.93	+0.63	+4.94	+3.80
0.87	0.40	805	290	148	72	810	295	152	75	+0.62	+1.72	+2.70	+4.17
0.91	0.50	699	252	128	63	695	250	135	65	−0.57	−0.79	+5.47	+3.37
0.93	0.45	505	182	93	45	510	185	92	45	+0.99	+1.65	−1.08	0.00
0.97	0.60	311	112	57	28	305	115	56	30	−1.93	+2.68	−1.75	+7.14

Use 1 – Prevalence for Specificity
